# FANCA Polymorphism Is Associated with the Rate of Proliferation in Uterine Leiomyoma in Korea

**DOI:** 10.3390/jpm10040228

**Published:** 2020-11-13

**Authors:** Eunyoung Ha, Seungmee Lee, So Min Lee, Jeeyeon Jung, Hyewon Chung, Eunsom Choi, Sun Young Kwon, Min Ho Cha, So-Jin Shin

**Affiliations:** 1 Department of Biochemistry, School of Medicine, Keimyung University, 56 Dalseong-ro, Jung-gu Daegu 41931, Korea; hanne.md@gmail.com; 2 Department of Gynecology and Obstetrics, School of Medicine, Keimyung University, 56 Dalseong-ro, Jung-gu Daegu 41931, Korea; seungmeemd@gmail.com (S.L.); hyewonny81@naver.com (H.C.); eunsom1113@naver.com (E.C.); 3 Clinical Research Division, Korea Institute of Oriental Medicine, 1672 Yuseeong-daero, Yuseong-Gu, Daejeon 34054, Korea; dasonya@kiom.re.kr (S.M.L.); jjy0918@kiom.re.kr (J.J.); 4 Department of Pathology, School of Medicine, Keimyung University, 56 Dalseong-ro, Jung-gu Daegu 41931, Korea; pathol72@dsmc.or.kr

**Keywords:** uterine leiomyoma, polymorphism, personalized medicine, FANCA

## Abstract

Uterine leiomyomas are the most common benign gynecologic tumors. This study was aimed to identify single nucleotide polymorphism of Fanconi anemia complementation group A (FANCA), associated with the rate of proliferation in uterine leiomyomas. In vitro study of patient-derived primary-cultured leiomyoma cells and direct sequencing of fresh frozen leiomyoma from each subject was conducted. Leiomyomas obtained from 44 patients who had underwent surgery were both primary-cultured and freshly frozen. Primary-cultured leiomyoma cells were divided into, according to the rate of proliferation, fast and slow groups. Single nucleotide polymorphism (SNP) of FANCA were determined from fresh frozen tissues of each patient using direct sequencing. Direct sequencing revealed a yet unidentified role of FANCA, a caretaker in the DNA damage-response pathway, as a possible biomarker molecule for the prediction of uterine leiomyoma proliferation. We identified that rs2239359 polymorphism, which causes a missense mutation in FANCA, is associated with the rate of proliferation in uterine leiomyomas. The frequency of C allele in the fast group was 35.29% while that in slow group was 11.11% (odds ratio (OR) 4.036 (1.176–13.855), *p* = 0.0266). We also found that the TC + CC genotype was more frequently observed in the fast group compared with that in the slow group (OR 6.44 (1.90–31.96), *p* = 0.0227). Taken together, the results in the current study suggested that a FANCA missense mutation may play an important regulatory role in the proliferation of uterine leiomyoma and thus may serve as a prognostic marker.

## 1. Introduction

Uterine leiomyomas, generally known as fibroids, are the most common benign gynecologic tumors. Approximately 20 to 40% of women of reproductive age are reported to have these benign tumors [1]. Although benign in their nature, leiomyomas are socioeconomically and clinically important, since 5 to 10% are estimated to be associated with infertility and 1 to 3% to cause infertility with other possible causes of infertility excluded [2]. With the increasing average age of women at the first childbirth, infertility has become a paramount issue both socioeconomically and clinically [3]. Due to the absence of uterus-sparing medical therapy, the treatment of choice of uterine fibroids is surgical removal via either hysterectomy or myomectomy. Patients with uterine leiomyomas, once diagnosed, have to go through regular check-ups for the progression of the growth of leiomyomas. Sometimes, leiomyoma would unexpectedly grow exponentially in the period of just a month, resulting in unwanted loss of fertility in women of reproductive age. Other times, it remains the same for years. Currently, no therapeutic tool that can predict the growth patterns of leiomyoma is available. Thus, the unpredictability of the growth patterns of leiomyoma is the utmost important unmet medical need regarding the treatment of uterine leiomyoma. Considering the high incidence of leiomyoma, absence of effective medical treatment, and its estimated contribution to infertility, the prognosis of leiomyoma, including growth rate and size variability, is of great importance for women of reproductive age.

Fanconi anemia complementation group A (FANCA), one of Fanconi anemia complementation group (FANC) family genes, codes for a protein that is part of the Fanconi anemia (FA) complex. FA complex mediates the FA pathway which maintains chromosome stabilities via interstrand DNA cross-link repair [4]. FA core complex proteins recruit DNA repair molecules, such as BRCA1, to the area of DNA damage so the error can be repaired. The FA pathway acts, using components of other known DNA repair processes, when DNA damages inhibit replication. Disorders or mutations in this gene cause an autosomal recessive disorder, the main features of which are aplastic anemia in childhood, multiple congenital abnormalities, susceptibility to leukemia and other cancers [4,5]. Evidence indicates that polymorphisms or mutations in the FANCA gene are related to cancer survival and susceptibility [6,7,8,9]. Among numerous polymorphisms of FANCA, rs2239359, a missense single nucleotide polymorphism (SNP) changing serine to glycine, is known to be associated with premature ovarian failure in Korean women [10].

In this study, we discovered the association of rs2239359 polymorphism with the rate of proliferation in leiomyoma in Korea.

## 2. Materials and Methods

### 2.1. Study Subjects

Uterine leiomyoma tissues were obtained from patients who underwent myomectomy or hysterectomy at Dongsan medical center (Daegu, Korea) between 2010–2017. Leiomyoma tissues from a total of 44 patients who did not receive preoperative hormonal therapy, including ulipristal acetate and GnRH agonist, were used in this study. Patients in the postmenopausal period were excluded. Written informed consent was obtained from all patients and the study was approved by the institutional review board of Dongsan Medical Center (Daegu, Korea; approval no. 09-156).

### 2.2. Isolation of Uterine Leiomyoma Cells and Culture

The tissue was washed twice in cold phosphate buffered saline before being minced into 5 mm pieces in a sterile culture dish. The minced tissue pieces were transferred into 50 mL conical tubes containing Hanks’s balanced salts (Sigma-Aldrich, St Louis, MO, USA) supplemented with 25 mM HEPES (4-(2-hydroxyethyl)-1-piperazineethanesulfonic acid), 100 units/mL antibiotics, 1.5 mg/mL collagenase IV (Sigma-Aldrich) and 0.2 mg/mL of DNase I (Roche Diagnostics, Mannheim, Germany). The tubes were kept at 37 °C in a water bath with gentle agitation for 3 h. Undigested tissues were filtered, and the cells were centrifuged at 1000 rpm for 5 min. The pellet was rinsed once with Hanks’s balanced salts and dispersed in a complete medium composed of DMEM (Dulbecco Modified Eagle Medium)/F12 with 10% fetal bovine serum, 1% penicillin/streptomycin. The cells from the second passage were used for the experiments. To assess doubling time, 3 × 10^5^ cells/well were plated in 100 mm dishes.

### 2.3. Doubling Time Measurement

Cells were seeded in 100 mm dishes, with 2 × 2 mm crossing lines drawn at the bottom, at a concentration of 10,000 cells/well with a medium containing 10% fetal bovine serum and 1% antibiotics. Doubling time was determined with the following formula: Doubling time (DT) = TIn2/In(Xe/Xb), where T is incubation time, Xb is the cell number at the beginning of the incubation, and Xe is the cell number at the end of the incubation. Primary-cultured leiomyoma cells were then divided into two groups, fast (DT < 144 h) and slow (DT > 144 h), according to doubling time.

### 2.4. Preparation of Genomic DNA and Identification of SNP

**Figure 1:**
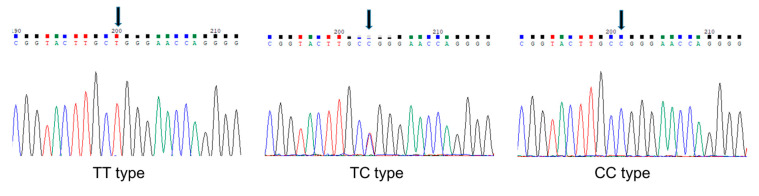
Polymorphic sites identified in the FANCA gene. Arrows indicate rs2239359 SNP.

Genomic DNA was isolated from leiomyoma tissue of each subject using GenExTM Tissue, Sx (Geneall, Seoul, Korea) according to the manufacturer’s protocol. The genotypes of rs2239359 SNP from each of subject were determined by SolGent Co., Ltd. (Daejeon, Korea), using a BigDye Terminator v3.1 cycle sequencing kit (Thermo Fisher Scientific, Seoul, Korea) and an ABI3730XL DNA analyzer (Thermo Fisher Scientific, Seoul, Korea). The primers for PCR and sequencing were CTCAAGCAACATTACCTCAG (forward) and CCATCTCCTGACCTCGTGAT (reverse) and the genotype was determined using Vector NTI-Contig Express (Thermo Fisher Scientific, Seoul, Korea). Hardy–Weinberg equilibrium (HWE) was evaluated by chi-square test using Haploview 4.2 (HUGO Gene Nomenclature Committee: http://www.genenames.org/ (accessed on 12 June 2018). Each of rs2239359 genotypes is shown in Figure 1.

### 2.5. Statistical Analysis

Data were analyzed with SPSS software, version 19.0 (IBM Analytics, Seoul, Korea). Differences in continuous variables were determined using parametric Student’s *t*-test. Categorical variables were compared with a chi-square test or Fisher’s exact test. The association of rs2239359 with the proliferation of leiomyoma was analyzed using binary logistic regression and adjusted for age, body mass index (BMI), smoking, drinking, recurrence, hypertension, and the presence of diabetes mellitus. Odds ratios (ORs) with 95% confidence intervals (95% CI) were calculated. To investigate whether the rs2239359 polymorphism is associated with the clinical parameters of the study subjects, we performed a statistical analysis using a general linear model adjusted for age, BMI, smoking, and drinking status. Statistical significance was set at *p* < 0.05.

## 3. Results

### 3.1. Anthropometrics, Comorbidities, and Blood Chemistry

The general anthropometric characteristics in the slow and fast groups are shown in Table 1. Body weight, height, and age were not different between the two groups. No differences in the number and size of leiomyomas were observed between the two groups. Also, the presence of comorbidities (hypertension, diabetes mellitus, thyroid disease, and hyperlipidemia) was not different. We did not find any association between factors in blood chemistry and the rate of proliferation of leiomyoma (Table 2). Of note, hematocrit showed a tendency to decrease in the fast group (*p* = 0.085).

### 3.2. Association of rs2239359 SNP in FANCA with the Proliferation Rate of Leiomyoma

To find the possible association between the missense SNP rs2239359 in FANCA and the proliferation rate of leiomyoma, we performed direct sequencing (Figure 1). The rs2239359 SNP in FANCA satisfied the Hardy–Weinberg equilibrium (HWE) (*p* = 0.658), and minor allele frequency (MAF) was 0.205, which was slightly lower than in a previous report [10] and NCBI information [11]. Table 3 shows the allele and genotype distributions of rs2239359 in the fast and slow groups. In the fast group, C allele frequency was 35.29%, which was significantly higher than that in the slow group (11.11%, OR = 4.036 (1.176–13.855), *p* = 0.0266). The frequency of subjects with C allele in the fast group was 64.7%, which was also higher than 22.2% of the slow group (OR = 6.44 (1.90–31.96), *p* = 0.023).

### 3.3. rs2239359 in FANCA and Thyroid Function

**Figure 2:**
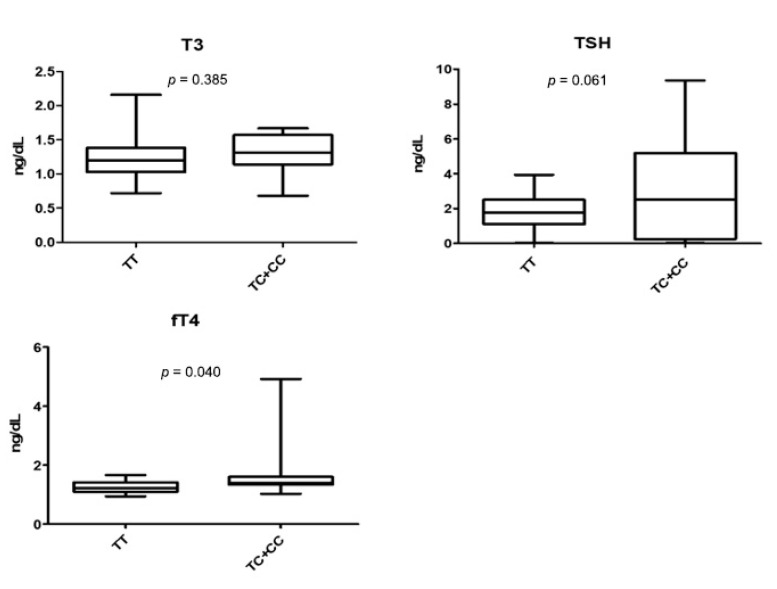
Values of thyroid functional markers (T3, TSH, and fT4) in TT and TC + CC genotypes of rs2239359 in FANCA.

Although we found no significant difference between thyroid functional markers (T3, TSH, and fT4) and the proliferation rate of leiomyoma (Table 2), we found an association of rs2239359 in FANCA with the serum fT4 level (Figure 2). The average serum fT4 level in subjects with C allele was 1.69 ng/dL, which was significantly higher than 1.26 ng/mL of subjects with the TT type (*p* = 0.040). TSH also showed an increased tendency in subjects with C allele (*p* = 0.061). T3 level showed no difference between the two groups. We observed no other association of rs2239359 in FANCA with any factors in blood parameters (Table 4).

## 4. Discussion

In this study, we found that FANCA missense SNP rs2239359 was associated with increased proliferation rate of leiomyoma in Korean women. We also found the association of rs2239359 with serum fT4 level.

FANCA is a major member of the Fanconi anemia (FA) core complex genes, mutations of which are responsible for most cases of FA, a heritable autosomal recessive disease characterized by high susceptibility to chromosomal breakage, leading to progressive bone marrow failure, congenital abnormalities, and cancer predisposition [12]. Clinical manifestations of FA are highly variable, implicating genetic heterogeneity. FANCA mutant mice exhibited growth retardation, microphthalmia, and craniofacial malformations [13]. One of the various clinical manifestations of FA is impaired fertility. Women with FA present hypoplastic ovaries with menstrual irregularities, secondary amenorrhea, anovulation, and premature senescence of reproduction [14,15]. Mutant FANCA mice displayed reduced ability to reproduce and gonadal defects [13,16,17].

Of the numerous clinical manifestations that are related to FANCA mutations, genetic association of FANCA mutations with uterine leiomyoma, especially in relation to the proliferation rate, has not been reported. Given the extent of genetic heterogeneity of the FANCA mutation and its involvement in diverse cellular functions, finding the association of FANCA missense SNP rs2239359 with the proliferation rate of leiomyoma in the current study was not surprising. To support a highly possible role of FANCA in the growth of leiomyoma, a study reported protein–protein interactions between FANCA and BRCA1 in leiomyomas [18]. FA core complex recruits key molecules of DNA repair system through the FA/BRCA pathway [9]. BRCA1 also participates in the homologous recombination process and double-strand break repair. Therefore, the loss of activity of FANCA could result in DNA damage and contribute to the differential growth rate of leiomyoma.

Further, a very recent study revealed that genes involved in genome stability are associated with the development of uterine leiomyoma [19]. They showed the association of ATM and TP53 with uterine neoplasia, possibly through genetic instability. Interestingly, like FANCA, ATM and TP53 are regulatory molecules involved in the DNA damage response [20]. They have also been found to be recurrently mutated in leiomyosarcoma [21].

FANCA is believed to induce the synthesis of GnRH [22], which in turn stimulates gonadotropin and sex hormones, including estrogen. Estrogen is a firmly established inducer of leiomyoma growth [23]. It also affects thyroid functions [24,25]. Thyroid dysfunction in patients with leiomyoma after administration of a GnRH agonist has been reported [26,27]. Given the result in this study and the above-mentioned information, there might be a possible, not yet identified relationship between FANCA and thyroid hormones in relation to the growth of leiomyoma. Further studies establishing the mechanistic details to determine the effect of FANCA on thyroid function in relation to the growth of leiomyoma will be of great importance.

This study has limitations such as sample size and absence of other SNP information positioned in FANCA. We also have not undertaken the diepoxybutane (DEB) and mitomycin-C (MMC) stress test to rule out FA. However, we excluded all the anthropomorphic associations among the patients to rule out FA. Nevertheless, we found the relation between rs2239359 of FANCA and proliferation of leiomyoma in Korean women. We also found the association of rs2239359 with the serum fT4 level. The significance of this study lies in the finding of FANCA SNP rs2239359 as the possible determining factor for the differential growth rate of leiomyoma. Since FANCA mutations are highly common in gynecological malignant tumors such as breast cancer, further studies including DEB and MMC stress test to determine the factors that render leiomyoma benign and biomarkers that predict the growth patterns of leiomyoma will be of great benefit.

## Figures and Tables

**Table 1:** Anthropometric and clinical characteristics of the slow and fast groups.

	Slow	Fast	*p * ^1^
N	26	16	
Anthropometric characteristics			
Age (years)	39.58 ± 7.22	42.75 ± 8.82	0.211
Weight (kg)	59.83 ± 7.74	58.36 ± 10.49	0.820
Height (cm)	158.28 ± 6.47	157.82 ± 6.13	0.606
BMI (kg/m^2^)	23.92 ± 3.20	23.45 ± 4.22	0.683
Smoking (None/Yes)	26/0	15/1	0.381
Drinking (None/Yes)	20/6	14/2	0.688
Myoma number	3.23 ± 3.02	2.88 ± 4.40	0.757
Myoma size (mm)	81.69 ± 24.26	92.06 ± 31.16	0.235
Disease history			
Recurrence of leiomyoma	2 (7.7%)	1 (6.3%)	1.000
Hypertension	1 (3.8%)	1 (6.3%)	1.000
Diabetes mellitus	0 (0.0%)	1 (6.3%)	0.381
Thyroid disease	2 (7.7%)	3 (18.8%)	0.352
hyperlipidemia	0 (0.0%)	1 (6.3%)	0.381
Previous operation	2 (7.7%)	1 (6.3%)	1.000

^1^*p* was calculated by chi-square test or student *t*-test.

**Table 2:** Association of blood parameters with the proliferation of leiomyoma.

	Slow	Fast	*p*
WBC (10^3^/μL)	5.32 ± 1.33	5.89 ± 1.70	0.139
RBC (10^6^/μL)	4.36 ± 0.33	4.27 ± 0.65	0.506
Hb (g/dL)	12.47 ± 1.30	12.06 ± 2.04	0.385
HCT (%)	38.57 ± 3.03	34.92 ± 6.34	0.085
MCV (fL)	89.02 ± 6.31	86.13 ± 7.28	0.209
MCH (pg)	28.65 ± 2.87	28.43 ± 3.65	0.825
MCHC (g/dL)	43.99 ± 58.62	32.89 ± 2.20	0.411
Platelet (10^3^/μL)	291.76 ± 68.19	324.60 ± 103.04	0.341
MPV (fL)	10.35 ± 13.71	7.64 ± 1.01	0.489
Neutrophil (%)	56.90 ± 9.40	58.53 ± 12.74	0.578
Lymphocyte (%)	32.69 ± 8.78	36.87 ± 17.60	0.333
Monocyte (%)	5.49 ± 1.22	5.67 ± 1.67	0.596
Eosinophil (%)	2.52 ± 1.61	2.75 ± 2.68	0.728
Basophil (%)	0.64 ± 0.30	0.54 ± 0.31	0.311
LUC (%)	1.76 ± 0.53	1.66 ± 0.58	0.614
aPTT (sec)	27.72 ± 1.68	27.61 ± 2.35	0.914
PT (INR)	1.02 ± 0.05	1.00 ± 0.05	0.574
Sodium (mmol/L)	138.48 ± 1.76	138.20 ± 2.21	0.988
Potassium (mmol/L)	4.08 ± 0.37	4.13 ± 0.27	0.794
Chloride (mmol/L)	104.76 ± 1.51	98.51 ± 24.33	0.258
Total Cholesterol (mg/dL)	197.76 ± 33.17	193.67 ± 23.54	0.606
Total calcium (mg/dL)	13.00 ± 18.54	9.43 ± 0.35	0.403
Phosphorus (mg/dL)	3.65 ± 0.46	3.75 ± 0.50	0.885
Glucose (mg/dL)	97.52 ± 14.12	102.67 ± 28.00	0.756
BUN (mg/dL)	12.96 ± 3.13	12.80 ± 5.20	0.641
Creatinine (mg/dL)	0.62 ± 0.08	0.69 ± 0.21	0.268
Total Protein (g/dL)	7.36 ± 0.39	7.58 ± 0.46	0.201
Albumin (g/dL)	4.42 ± 0.21	4.43 ± 0.18	0.911
Total Bilirubin (mg/dL)	0.57 ± 0.15	0.60 ± 0.17	0.494
ALP (U/L)	54.44 ± 15.66	60.00 ± 12.21	0.613
AST (U/L)	19.36 ± 5.31	20.20 ± 5.07	0.554
ALT (U/L)	13.40 ± 7.16	13.47 ± 5.01	0.756
eGFR (mL/min/1.73 m^2^)	108.66 ± 17.46	101.21 ± 24.21	0.459
T3 (ng/mL)	1.25 ± 0.30	1.29 ± 0.27	0.554
TSH (uIU/mL)	1.96 ± 1.09	3.05 ± 2.84	0.219
free T4 (ng/dL)	1.43 ± 0.76	1.36 ± 0.17	0.670

Note: WBC = white blood cell; RBC = red blood cell; Hb = hemoglobin; HCT = hematocrit; MCV = mean corpuscular volume; MCH = mean corpuscular hemoglobin; MCHC = mean corpuscular hemoglobin concentration; MPV = mean platelet volume; LUC = large unstained cells; aPTT = activated partial thromboplastin time; PT = prothrombin time; INR = international normalized ratio; BUN = blood urea nitrogen; ALP = alkaline phosphatase; AST = aspartate aminotransferase; ALT = alanine aminotransferase; eGFR = estimated glomerular filtration rate; T3 = triiodothyronine; TSH = thyroid stimulating hormone; T4 = thyroxine.

**Table 3:** Distribution of allele and genotype of rs2239359 in FANCA in the slow and fast groups.

		Slow Group	Fast Group	OR (95% CI)	*p*
allele					
	T	48 (88.89%)	22(64.71%)	4.04 (1.18–13.86)	0.0266
	C	6 (11.11%)	12(35.29%)		
genotype					
	TT	21 (77.8%)	6 (35.3%)	6.44 (1.90–31.96)	0.0231
	TC+CC	6 (22.2%)	11(64.7%)		

**Table 4:** Association of rs2239359 genotype in FANCA with blood parameters in subjects with leiomyomas.

	TT	TC + CC	*p*
WBC (10^3^/μL)	5.57 ± 1.49	5.47 ± 1.53	0.897
RBC (10^6^/μL)	4.31 ± 0.51	4.35 ± 0.40	0.347
Hb (g/dL)	12.45 ± 1.66	12.09 ± 1.54	0.884
HCT (%)	37.50 ± 5.20	36.70 ± 4.26	0.737
MCV (fL)	88.74 ± 5.88	86.60 ± 8.03	0.368
MCH (pg)	28.90 ± 2.62	28.02 ± 3.89	0.334
MCHC (g/dL)	44.25 ± 58.56	32.47 ± 2.48	0.244
Platelet (10^3^/μL)	297.00 ± 83.72	315.87 ± 84.01	0.507
MPV (fL)	10.36 ± 13.71	7.62 ± 1.09	0.542
Neutrophil (%)	56.06 ± 7.44	59.92 ± 14.52	0.226
Lymphocyte (%)	33.64 ± 7.06	35.28 ± 19.14	0.754
Monocyte (%)	5.50 ± 1.21	5.65 ± 1.67	0.799
Eosinophil (%)	2.42 ± 1.53	2.90 ± 2.74	0.920
Basophil (%)	0.63 ± 0.31	0.55 ± 0.30	0.494
LUC (%)	1.74 ± 0.48	1.69 ± 0.67	0.519
aPTT (sec)	27.87 ± 1.76	27.37 ± 2.21	0.922
PT (INR)	1.02 ± 0.05	1.00 ± 0.03	0.485
Sodium (mmol/L)	138.52 ± 1.73	138.13 ± 2.23	0.937
Potassium (mmol/L)	4.04 ± 0.26	4.21 ± 0.42	0.151
Chloride (mmol/L)	104.84 ± 1.31	98.38 ± 24.31	0.335
Total Cholesterol (mg/dL)	198.68 ± 31.88	192.13 ± 26.08	0.481
Total calcium (mg/dL)	13.02 ± 18.54	9.39 ± 0.36	0.261
Phosphorus (mg/dL)	3.61 ± 0.44	3.82 ± 0.52	0.428
Glucose (mg/dL)	97.16 ± 15.09	103.27 ± 26.98	0.724
BUN (mg/dL)	13.12 ± 3.19	12.53 ± 5.11	0.728
Creatinine (mg/dL)	0.64 ± 0.09	0.66 ± 0.21	0.707
Total Protein (g/dL)	7.35 ± 0.39	7.61 ± 0.45	0.076
Albumin (g/dL)	4.40 ± 0.19	4.47 ± 0.21	0.204
Total Bilirubin (mg/dL)	0.57 ± 0.16	0.60 ± 0.16	0.417
ALP (U/L)	55.60 ± 16.68	58.07 ± 10.44	0.666
AST (U/L)	19.12 ± 4.59	20.60 ± 6.09	0.869
ALT (U/L)	13.36 ± 7.42	13.53 ± 4.31	0.509
eGFR (mL/min/1.73 m^2^)	106.60 ± 17.28	104.65 ± 25.13	0.858

*p* was calculated using a general linear model and adjusted for age, BMI, smoking, and drinking. Note: WBC = white blood cell; RBC = red blood cell; Hb = hemoglobin; HCT = hematocrit; MCV = mean corpuscular volume; MCH = mean corpuscular hemoglobin; MCHC = mean corpuscular hemoglobin concentration; MPV = mean platelet volume; LUC = large unstained cells; aPTT = activated partial thromboplastin time; PT = prothrombin time; INR = international normalized ratio; BUN = blood urea nitrogen; ALP = alkaline phosphatase; AST = aspartate aminotransferase; ALT = alanine aminotransferase; eGFR = estimated glomerular filtration rate.
